# Supplementary material for: Scattering Correction through Fourier-Domain Intensity Coupling in Two-Photon Microscopy (2P-FOCUS)
Source: ArXiv. 2025 Feb 10:arXiv:2401.15192v2. Preprint. [Version 2] (PMC11844625)
Supplement: Supplement 1 [file NIHPP2401.15192v2-supplement-1.pdf]

Appendices

Appendix A 2P-FOCUS Setup

The laser source for 2P-FOCUS is a femtosecond pulsed laser at 1035 nm wavelength and 1 MHz repetition rate (Monaco 1035-40-40 LX, Coherent). The maximum power used for 2P-FOCUS is 2.4 W (The maximum total power of the laser is 40 W but 37.6 W is used to pump an optical parametric amplifier, which is not used for 2P-FOCUS). A polarizing beam splitter cube (PBS123, Thorlabs) and a half-wave-plate (WPHSM05-1310) mounted on a rotation mount (PRM05, Thorlabs) are used to manually adjust the input power to the following optics in the system. A 2-axis galvo-mirror system (GVS002, Thorlabs) are placed on the Fourier plane to scan the laser beam in 2D for imaging in Fig. 4 and Fig. 5. The laser beam is then expended with a 4-f system (L1, LA1401-B, f_1 = 60 mm, Thorlabs; L2, AC508-100-C-ML, f_2 = 100 mm, Thorlabs). Next, the laser beam is pre-dispersed by a ruled grating (GR13-0310, 300/mm, 1000 nm blaze, Thorlabs) on the Fourier plane to compensate for the dispersion induced by the DMD. After the

grating, the pre-dispersed beam is relayed to the DMD with a 4-f system (L3, LB1199-C, f_3 = 200 mm, Thorlabs; L4, AC508-200-C-ML, f_4 = 200 mm, Thorlabs). The DMD (DLP650LNIR, 1280×800 pixels, maximum pattern rate 12.5 kHz, VIALUX) is placed on the Fourier plane to project binary intensity masks. The beam from the DMD is relayed to the back aperture of the objective lens by two 4-f systems, which is simplified as one 4-f system in the optical schematic diagram in Fig. 1D. The first 4-f system is to expend the beam (AC508-150-C-ML, f = 150 mm, and LA1256-C, f = 300 mm). The second 4-f system is to 1:1 relay the beam (two AC508-200-C-ML, f = 200 mm). A dichroic mirror (FF880-SDi01-t3-35x52, Semrock) is used to reflect the beam to the back-aperture of the objective lens (XLUMPlanFL N, 20×, 1.00 NA, water immersion, Olympus). Samples are placed on a manual 3-axis translation stage (MDT616, Thorlabs). In the emission path, a shortpass filter (ET750sp-2p8, CHROMA) is used to block the reflected excitation light. A bandpass filter (AT635/60m, CHROMA) is used in the experiments for Fig. 2 and Fig. 4 to pass through red fluorescence, while it is removed in the experiments for Fig. 5 because the emission wavelength of FITC-dextran is 520 nm. The fluorescence is detected by a PMT (H15460, Hamamatsu). A sCMOS camera (Kinetix22, Teledyne Photometrics) is used to visualize the focus in Fig. 2F-H. A beam turning cube (DFM1-E02, Thorlabs) is used to switch between the camera and the PMT. In addition, a one-photon widefield microscope is implemented, overlapping with 2P-FOCUS, to locate the sample and find the focal plane before two-photon imaging. The one-photon system consists of a LED (M565L3, Thorlabs), an aspherical condenser lens (ACL25416U-A) to collimate the LED light, and a dichroic mirror (AT600dc, CHROMA) to combine the one-photon path to the two-photon path. A power meter (PowerMax USB - PM10-19C Power, Coherent) is used to measure laser power. During imaging sections, the illumination power on the sample is measured at a relayed image plane located before the objective lens but after the DMD. This measurement is then multiplied by the power loss rate due to the optical parts situated between the relayed image plane and the sample.

The 2P-FOCUS system is controlled by a computer (OptiPlex 5000 Tower, Dell) using MATLAB and a data acquisition card (PCIe-6363, X series DAQ, National Instruments) for signal input/output. Voltage signals for externally triggering the laser and the DMD, as well as for controlling the scanning location, are generated using MATLAB and delivered to the devices by the DAQ card. Simultaneously, the fluorescence intensity detected by the PMT is read by the DAQ card through an analog input port.

Appendix B Dispersion Control

Dispersion control has been implemented when using a DMD to modulate femtosecond laser light [47]. The pixels on the DMD tilt to +12° or −12° along the diagonal direction of the pixels when projecting binary patterns, thereby inducing dispersion to the input excitation beam like a blazed reflective grating. This process can be described by the grating equation:

$$\Delta = d(sin\theta_i + sin\theta_m) = m\lambda, \tag{10}$$

where Δ denotes optical path difference, d denotes the spacing between grooves, θ_i denotes the incident angle, θ_m denotes the diffraction angle, m is the order of principal maxima, and λ denotes the wavelength of light (λ = 1035 ± 5 nm). The effective spacing between grooves of the DMD is calculated when the DMD is operated in the Littow configuration, that is, $\theta_i = \theta_m = 12°$ and $m = 1$. Therefore, the effective spacing between grooves of the DMD, d_{DMD}, is 2.49 μm, which is different from the pitch size (10.8 μm) of the DMD.

To compensate the angular dispersion induced by the DMD ($\mathfrak{D}_{DMD}$), we implemented a grating on the conjugate Fourier plane with a 4-f relay system (L3, L4 in Fig. 1D) to the DMD. The angular dispersion induced by the grating, $\mathfrak{D}_G$, satisfies:

$$\frac{\mathfrak{D}_G}{\mathfrak{D}_{DMD}} = \frac{f_4}{f_3}, \tag{11}$$

where the anglular dispersion is calculated as

$$\mathfrak{D} = \frac{m}{d\cos\theta_m}. \tag{12}$$

f_3 and f_3 are the focal length of lens L3 and L4, respectively. Considering the effective grooves (1000/2.49 = 402 grooves/mm) and the size of the DMD (13.8 × 8.6 mm^2), a grating with 300 grooves/mm and a 1:1 relay system are selected. The output beam from the DMD is designed to be perpendicular to the DMD, that is, $\theta_m = 0$. According to Eq. 10, 11, 12, when $m = 1$, the dispersion angle of the incident beam (λ = 1035 ± 5 nm) is 0.25°. Correspondingly, when $f_3 = f_4 = 200$ mm and $m = 1$, the incident angle to the grating is 25.1°. After the dispersion control, the focus becomes a symmetric circular spot (Fig. 1E).

Appendix C Data Processing

C.1 Process data acquired by the PMT.

Data are acquired for 1 ms per random pattern using the PMT in the first step of scattering correction. Within the 1 ms time frame, the laser is turned on for 0.9 ms and off for 0.1 ms. The measurements acquired while the laser is on are subtracted from the measurements acquired while the laser is off. After subtraction, all negative voltages are set to zero. Next, a box filter with a width of 3 pixels is applied to the time-lapse signal to remove fluctuations. The final value of the PMT signal for one random pattern modulation is the sum of the processed time-lapse voltage signals acquired over 0.9 ms. Repeating this process for PMT data acquired under all random patterns generates Fig. 2B.

C.2 Generate a correction mask from the processed PMT data.

The processed PMT data is sorted, and the random patterns corresponding to the top 10% brightest data are selected. The sum of the selected random patterns generates Fig. 2C.

C.3 Binarize the grayscale correction mask.

The intensity distribution on the DMD is measured with a fluorescence slide without scattering media before every experiment, and is referred to as an intensity calibration map $I_{map}(f_x, f_y)$. The intensity calibration map is measured by turning on one super-pixel at a time and recording the corresponding fluorescence intensity. The laser power at the image plane, P_0, is also measured when all pixels are turned on. Therefore, the laser power P of any intensity mask $M(f_x, f_y)$ (a random mask or a correction mask) can be calculated by $P = P_0 \sum_{f_x=1}^{800} \sum_{f_y=1}^{800} M(f_x, f_y) \cdot I_{map}(f_x, f_y)$. By adjusting P_0 and/or $M(f_x, f_y)$, the laser power on the sample P is kept the same before and after correction. The largest improvement is achieved when P_0 is maximized and $M(f_x, f_y)$ is highly selective. In this case, P_0 is measured when the input power to the DMD is maximized by turning the half-wave plate. With the known values of P_0, I_{map}, and P, the threshold for binarizing the grayscale correction mask is calculated accordingly.

C.4 Generate and process 2D images

An image acquired by the PMT (Fig. 3—5) is generated by reassign the processed PMT data to their corresponding scanning locations. The first step is to remove the background from the image by subtracting the mean of 300 pixels with the lowest intensity, and setting all negative intensities to zero. Next, a 2×2 median filter is applied to the image to remove salt-and-pepper noise.

For images acquired by the camera (Fig. 2F-G), the background is removed by subtracting the images with a background image, and then a 3×3 median filter is applied to remove noise.

C.5 Generate the volumetric view of 3D image stack.

Fig. 4A and Fig. 5A are generated using ImarisViewer 10.1.0. The 3D image stacks consist of processed 2D images. The image stack is interpolated by 10 times along the z-axis for a better 3D display.

Appendix D Sample Preparation

D.1 Homogeneous fluorescence slide with bone for Fig. 2

A microscope slide (CAT. NO. 3049, Gold Seal) is coated with a thin layer of fluorescent paint (Tamiya color, fluorescent red). After the paint is dry, a piece of chicken bone is glued on it as the scattering medium using clear gorilla glue.

D.2 Fluorescence beads with bone for Fig. 3

Red fluorescence beads suspension (R700, Thermo Fisher Scientific, MA) is mixed with PDMS (Sylgard 184, Dow Inc, MI) in the ratio of 1:550. Base elastomer and curing agent of PDMS are mixed in the ratio of 10:1. After mixing, we use a vacuum desiccator to remove the air bubbles in the mixture for 30min. Then the mixture is poured onto a clean microscopy slide, covered by a coverslip, and heated at 100 Celsius for 35min to cure the PDMS. After the fluorescent beads are fixed, a piece of chicken bone is glued on top of it as the scattering medium using clear gorilla glue.

D.3 Whole brain preparation for Fig. 4

The PV tdTomato mouse (PV-IRES-Cre;LSL-tdTomato (Ai9)) was weighed and put into 5% isoflurane for initial induction. An IP injection of Ketamine hydrochloride 40-80 mg/kg + Xylazine 5-10 mg/kg was given and then the animal was put back into the isoflurane until the animal's breathing ceased. The animal was brought to the chemical fume hood and placed in the supine position. An additional amount of isoflurane was placed in a 10 cc syringe with a gauze and placed over the mouse's nose for additional anesthesia. The hair on the ventral thorax was soaked with 70% alcohol. The anesthetic depth was checked via lack of toe pinch response. A midline incision was made through the skin over the proximal abdomen and thorax. The skin was dissected to expose all underlying muscle. A cut was made into the abdomen, the diaphragm was punctured and a thoracotomy was made by bilateral para- midline incisions through the ribs toward the thoracic inlet, exposing the thoracic viscera. The catheter was placed in the left ventricle, the right atrium was cut to exsanguinate the mouse and allow for drainage of the perfusate. The animal was perfused with cold 4% paraformaldehyde, approximately 12 mls. The brain was dissected from the skull and then placed in cold 4% paraformaldehyde.

D.4 Fluorescein isothiocyanate-dextran (FITC-dextran) injection and whole tissue preparation for Fig. 5

Anesthesia was induced in mice with 2.0-3.0% isoflurane and maintained at 1.5-2.0%. Depth of anesthesia was monitored by toe-pinch and body temperature was maintained by a water perfused thermal pad (Gaymar T/Pump) set at 37°C. Fluorescein isothiocyanate-dextran (FITC-dextran) (MW = 2 MDa; Sigma-Aldrich) was injected retro-orbitally at a total volume of 150 μL. After retro-orbital injection, FITC-dextran was allowed to circulate for a total of 10-15 minutes and the animal was sacrificed. After the animal was sacrificed, the whole brain was collected and stored overnight in phosphate-buffered saline at 4°C and was imaged the next day.

	Principle of scattering correction	Time to complete the correction process	FOV after correction	Maximum imaging depth	Excitation light	Fluorescence label	Maximum improvement
**IMPACT Ref.23**	Wavefront compensation: In each iteration, half of the MEMS phase elements are modulated simultaneously, each at a unique frequency, while the other half remain stationary. Light from these two parts interferes.	The measurement time is ~5 s for 3 iterations. The time for computing and projecting phase masks is not mentioned.	~10 μm	400 μm ex vivo (460 μm in vivo in the mouse lymph node)	80 MHz, 920 nm, 24 mW - 180 mW	Layer 5 neurons with GFP	20 folds
**F-SHARP *Ref. 24**	Wavefront compensation: Splitting the excitation light into two beams and probing the scattered E-field PSF through the interference of these beams.	The correction time for brain imaging is not specified.	~85 μm	480 μm below dura in vivo without skull.	80 MHz, 920 nm, 25 mW	GAD67-GFP	5 folds
**DASH Ref. 25**	Wavefront compensation: Split the excitation light into two beams and update phase masks based on their interference. This method is compared in detail with IMPACT and F-SHARP.	It takes 10 min to complete the correction process for Fig. 3 mouse brain imaging. With a faster SLM, the measurement time can be reduced to 45 s. The time for computing and projecting the mask is not mentioned.	~30 μm	530 μm ex vivo (hippocampus tissue)	80 MHz, 900 nm, 25 mW	Microglia with GFP	7 folds
**ALPHA-FSS* Ref. 26**	The basic physical principle is the same as F-SHARP. The innovation compared to F-SHARP is direct focus sensing with phase-sensitive detection and conjugate phase using remote focusing. **Three-photon microscopy.**	3 s for measuring E-field PSF through cranial window. The time of computing and projecting mask is not specified.	~60 μm	3P imaging. 1140 μm below pia through cranial window in vivo.	1 MHz, 1300 nm. 15 mW-75 mW	Thy1-GFP	9 folds
**2P-FOCUS (Ours)**	Amplitude modulation on the back aperture of the objective lens couples light into naturally existing open channels in the scattering tissue. Open channels are probed by projecting binary random patterns, with no iteration.	Global correction takes 3 s, including 2.5 s for measurements, 0.5 s for computing the mask, and 1 ms for projecting the mask. For 9 subregions, the total time is 27 s (3x9).	230 μm	510 μm below most superficial blood vessel in a whole brain ex vivo.	1 MHz, 1035 nm, 2 mW-19 mW	Cell-fill tdTomato in PV neurons. FITC-dextran intravenous injection.	30.6 folds

Fig. S1. Table S1. Comparison between state-of-the-art multiphoton microscopy systems using active scattering correction and the 2P-FOCUS system. The reference numbers in the table refer to the bibliography in the main text.

Appendix E Supplementary Tables and Figures

All methods are based on two-photon microscopy except ALPHA-FSS [25], which is a three-photon microscopy system. The parameters listed in the table are collected from mouse brain images. $*$ These two works also demonstrate imaging through the skull in vivo, in addition to imaging through a cranial window. When imaging through the skull, F-SHARP [23] reaches a depth of 325 μm through a 50 μm-thick skull, and ALPHA-FSS [25] reaches a depth of 780 μm through a 100 μm-thick skull.

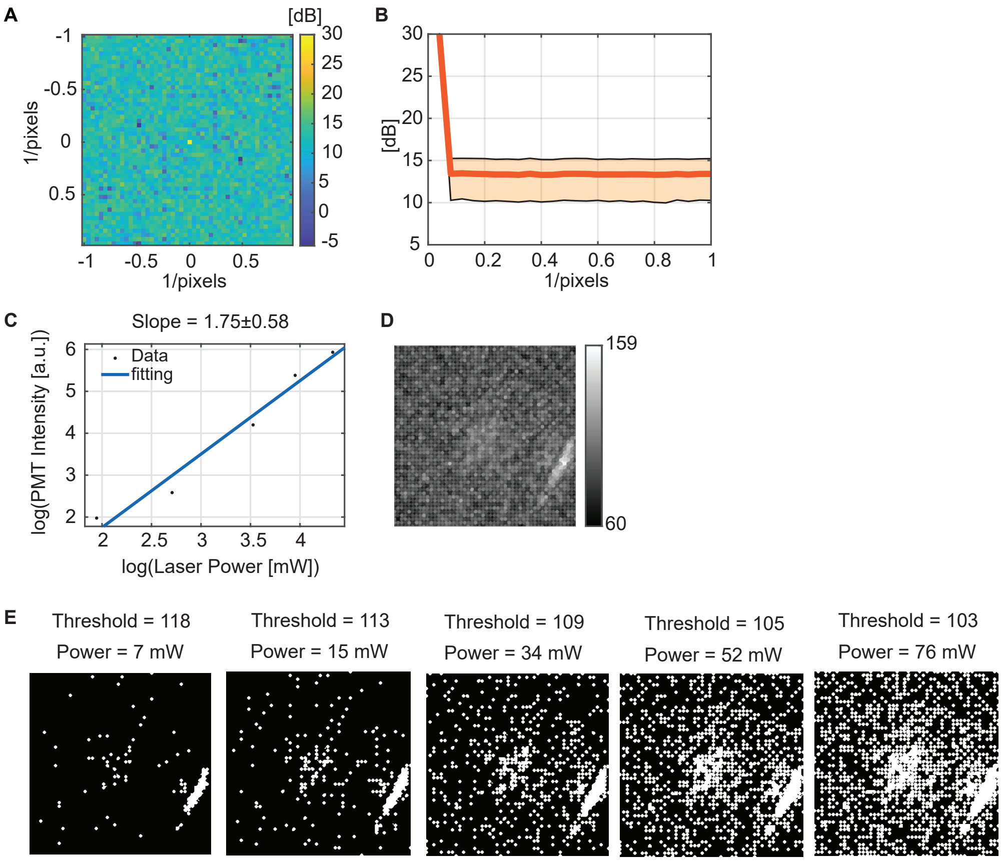

Fig. S1. The influence of the binarization threshold on correction masks. (A-B) Frequency analysis of 2,500 random patterns with a super-pixel size of 8 pixels and a sparsity of 0.4. (A) The 2D frequency spectrum of a single random pattern from the set. (B) The average frequency spectrum along the x-axis across the 2,500 random patterns. The red line represents the mean value, and the orange shading indicates the standard deviation. (C) Log-log plot of the fluorescence intensity before correction as a function of the laser power on the sample. The slope of the log-log plot is 1.75±0.58, indicating that the fluorescence intensity before correction increases quadratically with the illumination power. (D) The grayscale correction mask, the same as in Figure 2C. (E) The binary correction masks generated by applying different thresholds to (B). These masks are used to produce the data for “after correction" in Figure 2I.

Fig. 2J	Baseline	Sparsity 0.05	Sparsity 0.1	Sparsity 0.2	Sparsity 0.3	Sparsity 0.4
Mean	7.21	12.02	66.24	101.15	191.61	331.23
STD	0.75	1.22	3.28	3.72	5.29	7.29
Ratio	1	1.67	9.19	14.03	26.58	45.95

Table S2. The dataset from Fig. 2J and the corresponding improvement ratios.

Fig. S2D	Baseline	Sparsity 0.2	Sparsity 0.3	Sparsity 0.4	Sparsity 0.5
Mean	14.42	143.68	236.89	296.05	221.11
STD	3.26	6.28	7.56	10.99	8.80
Ratio	1	9.97	16.43	20.54	15.34

Table S3. The dataset from Fig. S2D and the corresponding improvement ratios.

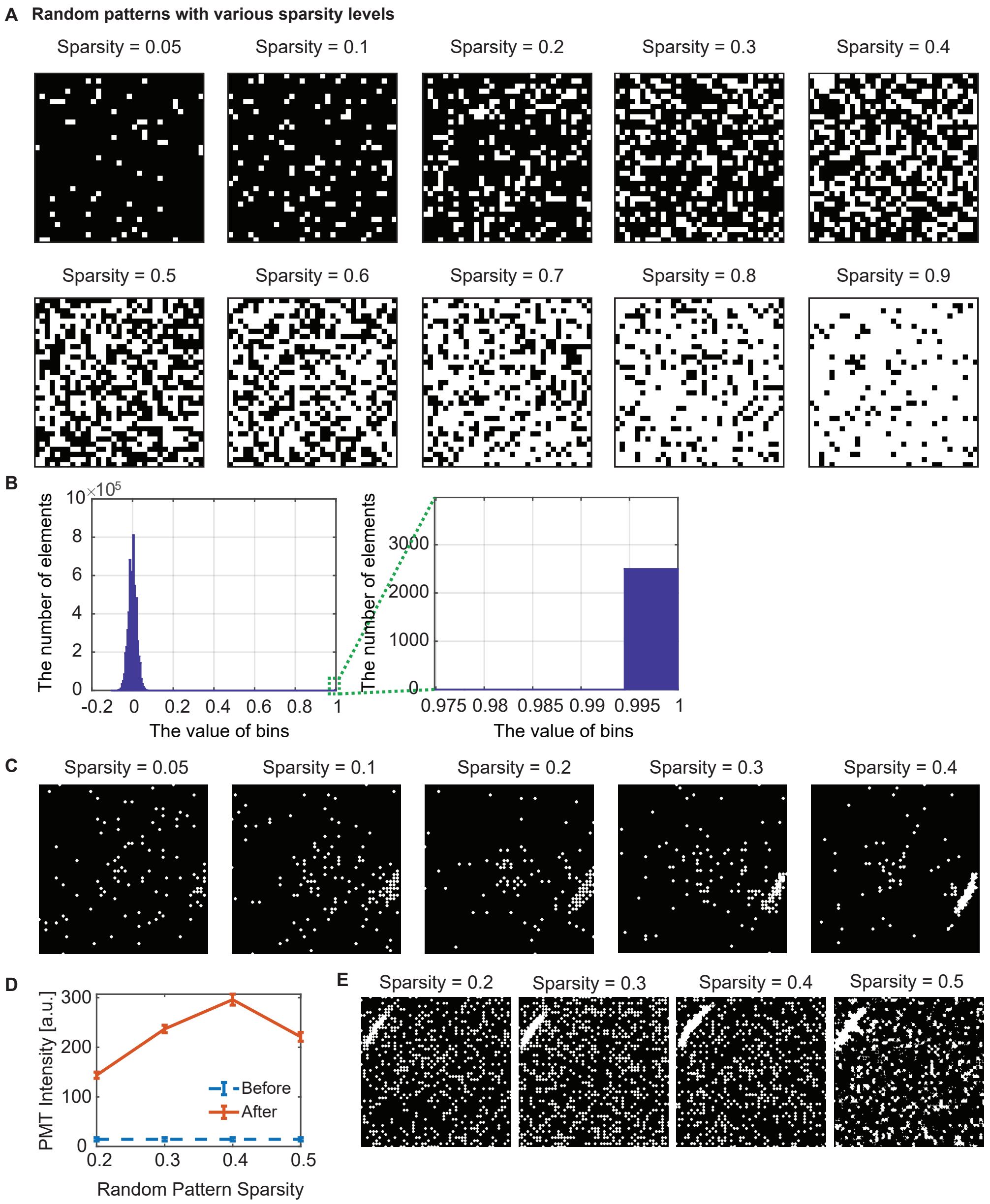

Fig. S2. The influence of the sparsity of random patterns on correction masks. (A) Random patterns with varying sparsity levels. Sparsity refers to the percentage of pixels that are turned on out of the total number. When the sparsity is between 0.5 and 0.9, the random patterns act more like notch filters, determining which pixels should be turned off rather than on. (B) Histogram of all elements in the matrix $I = P^T P$ (left) and a zoomed-in view (right). The zoomed-in view highlights the values of the diagonal elements in matrix I, while the other bins represent the non-diagonal elements. The results indicate that the dot product of any two distinct vectors in matrix P is 0 ± 0.02, and the dot product of a vector with itself is 1.

(C) Five correction masks used to generate the “after correction" data in Figure 2J. (D) Fluorescence intensity before (blue dashed line) and after (red line) correction as a function of random pattern sparsity. Data were collected by focusing a beam through another region of the bone. The results show that the fluorescence intensity decreases when the correction mask is generated with random patterns having a sparsity of 0.5 compared to those with a sparsity of 0.4. (E) Four correction masks used to generate the “after correction" data in (D) from random patterns with various sparsity levels.

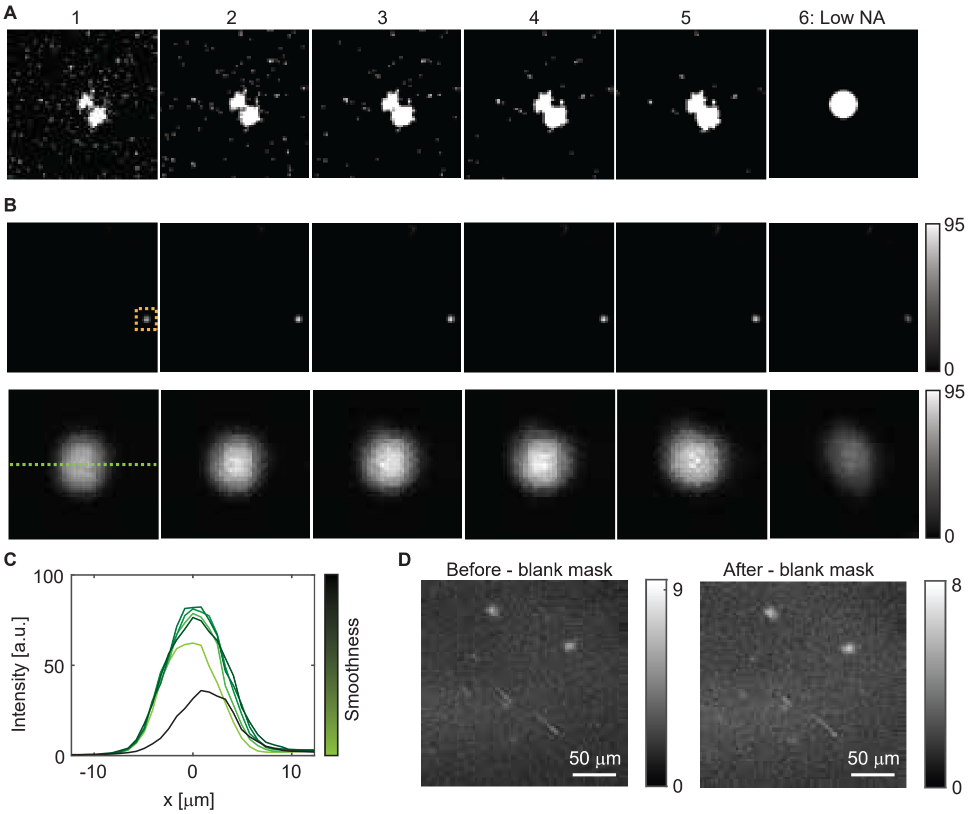

Fig. S3. (A) Correction masks with varying degrees of smoothing (plots 1–5), as well as a low-NA mask (plot 6). These masks provide the same output power given the same input power to the DMD. (B) Imaging of a red fluorescent bead through a piece of chicken bone under the intensity modulation of the corresponding masks. The second row shows a zoomed-in view of the region highlighted by the yellow box in the first row. All images are displayed using the same color scale. The maximum intensity is 95, achieved with the 4th correction mask, while the peak intensity under the low-NA mask is 42. (C) Intensity profile of the cross-section along the green dashed line in (B). (D) Examining photobleaching due to correction processes. The peak intensity decreased from 9 to 8 when comparing the image taken before the process without correction and the image taken after the process without correction.

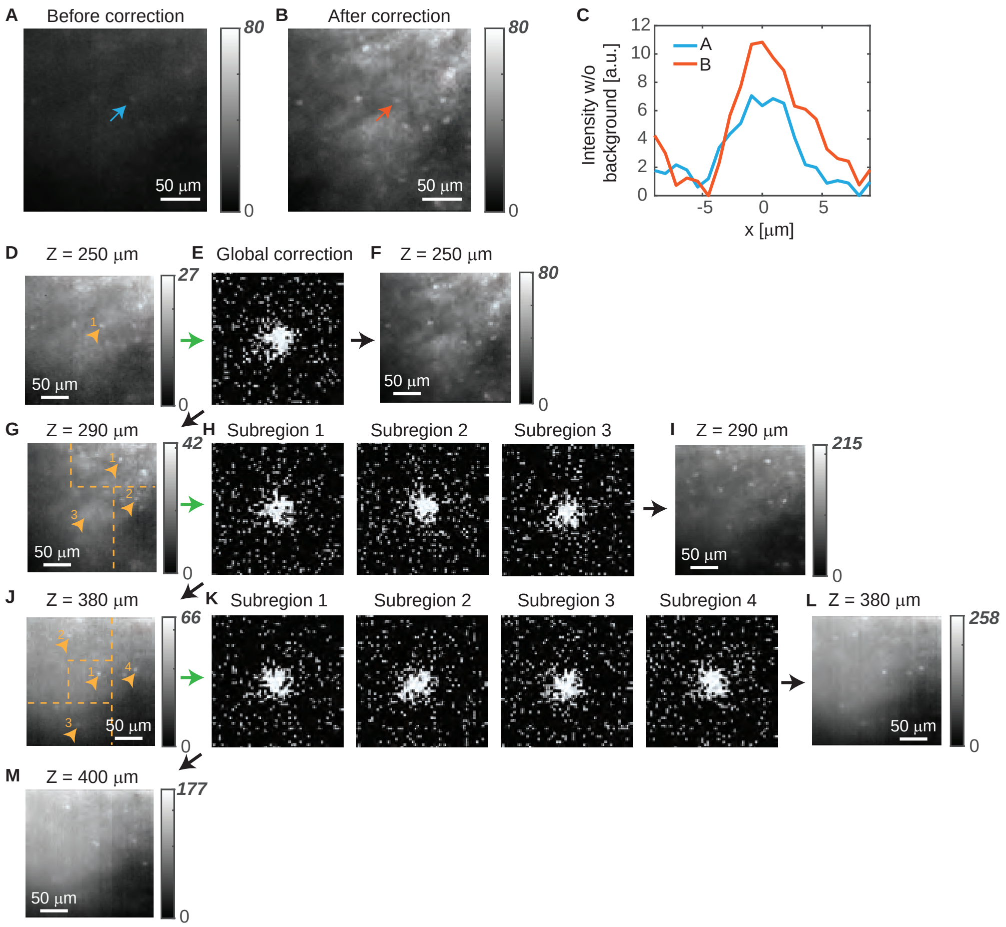

Fig. S4. Scattering correction for imaging PV neurons deep in the mouse brain. (A-C) Comparison of the images taken (A) before and (B) after global correction under the same color bar. The images are the same as in Figure 4E-F. The neuron used as a reference (pointed out by the arrows) becomes brighter rather than dimmer after correction. (C) The intensity profile of the reference neuron before and after correction. Background intensity is subtracted. The peak intensity of this neuron is improved by about 1.8-fold, which is not as much as the brightest neurons. (D-M) All correction performed for taking the $0-450\mu m$ image stack in Figure 4. The green arrows indicate the image is used to produce the corresponding mask. The black arrows point at images after correction. The yellow arrows in the images point to fluorescence objects used as references. Notice that (G), (J), and (M) are taken with last correction mask rather than blank screen on the DMD. (D-F) The first scattering correction is performed at 250 μm depth. (D) The image before correction, which is the same as Figure 4E. (E) A single correction mask is produced, which is the same as the bottom plot in Figure 4D. (F) The image after correction, which is the same as Figure 4F. (G-I) The second scattering correction is performed at 290 μm depth. (G) Image taken with the global correction mask in (E). The field-of-view is divided into three subregions. Notice that we used the background fluorescence as the reference in the third subregion to generate the correction mask. (H) Three correction masks corresponding to the three subregions. (I) Image after subregion correction. (J-L) The third scattering correction is performed at 380 μm depth. (J) Image taken with the three correction masks in (H). Four neurons are identified as the references for four subregions. (K) Four correction masks (identical to Figure 4J) corresponding to the four subregions. (L) Image after correction. (M) Image taken with the four correction masks (identical to Figure 4I) in (K) at 400 μm depth.

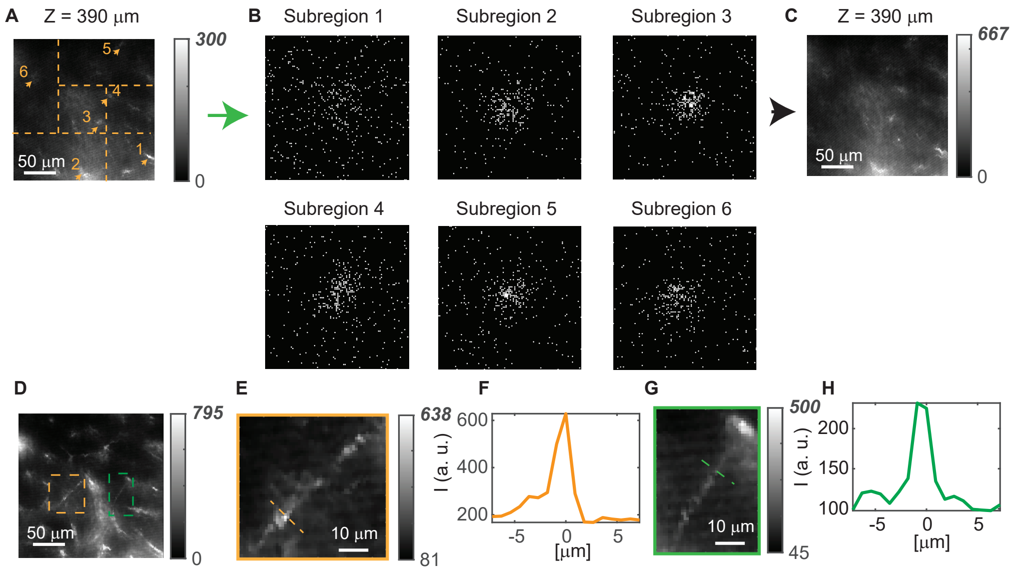

Fig. S5. Subregion correction is performed at 390 μm depth. (A) Image taken with the four correction masks in Fig. 5, identifying six fractions of blood vessels as references for six subregions. (B) Six correction masks corresponding to the six subregions. (C) Image after correction. (D) The same as Fig. 5E. (E, G) Zoomed-in views of capillaries in the boxed regions in (D). (F, H) Intensity profiles of the cross-sections of the capillaries in (E) and (G), marked by the dashed lines. Fine capillaries are resolvable after applying the correction masks.
